# Assessment of sublingual microcirculation for the screening of diabetic nephropathy

**DOI:** 10.1186/s13098-022-00864-3

**Published:** 2022-07-06

**Authors:** Qiang Li, XiaoXiao Liu, Mengxiao Jia, Fang Sun, Yingsha Li, Hexuan Zhang, Xiaoli Liu, Hongbo He, Zhigang Zhao, Zhencheng Yan, Zhiming Zhu

**Affiliations:** Center for Hypertension and Metabolic Diseases, Department of Hypertension and Endocrinology, Daping Hospital, Army Medical University, Chongqing, 400042 China

**Keywords:** Sublingual microcirculation, Type 2 diabetes mellitus, Urinary albumin creatinine ratio, Diabetic nephropathy

## Abstract

**Objective:**

To investigate the potential of employing sublingual microcirculation as an early noninvasive screening technique for diabetic nephropathy (DN).

**Research design and methods:**

We recruited 89 patients with type 2 diabetes mellitus (T2DM) and 41 healthy subjects in this cross-sectional observational study. All participants underwent fluorescein fundus angiography, vibration perception testing, 10 g (Semmes–Weinstein) monofilament examination, nerve conduction velocity, and 24-h urine microalbumin determination. HbA1c, fasting plasma glucose, blood lipid, and estimated glomerular filtration rate(eGFR) were measured. Sublingual microcirculatory images were captured using side-stream dark-field (SDF) microcirculation microscopy, and total and perfused vascular density (TVD and PVD) were calculated.

**Results:**

The sublingual microcirculatory parameters denoting microvascular density and perfusion were negatively correlated with both fasting plasma glucose (TVD, r =  − 0.316, *P* < 0.001; PVD, r =  − 0.350, *P* < 0.001; PPV, r =  − 0.279, *P* = 0.001) and HbA1c (TVD, r =  − 0.367, *P* < 0.001; PVD, r =  − 0.423, *P* < 0.001; PPV, r =  − 0.399, *P* < 0.001). Diabetes patients already had a reduction in sublingual microcirculation compared with healthy control, and more severe reductions in TVD (7.07 ± 1.64 vs. 9.67 ± 1.94 mm/mm^2^, *P* < 0.001) and PVD (5.88 ± 1.82 vs. 8.64 ± 2.46 mm/mm^2^, *P* < 0.001) were found in those diabetes patients developed microvascular complications. Sublingual microcirculation impairment was accompanied with higher urinary albumin creatinine ratio (UACR). Receiver operating characteristic (ROC) analysis showed that TVD (area under the curve, AUC = 0.890 [0.836 0.944], *P* < 0.001) and PVD (AUC = 0.883 [0.826, 0.940], *P* < 0.001) could be indicators for DN screening. We derived a combined predictor index (CPI) considering both TVD and PVD for screening DN, and both the AUC (0.892, [0.838 0.945], *P* < 0.001) and cutoff point of 11.30 mm/mm^2^ showed great improvement (sensitivity: 95.5%, specificity: 67.4%).

**Conclusions:**

Diabetes patients experienced impaired sublingual microcirculation, which was closely correlated with UACR. Sublingual microcirculation monitoring could be used for the noninvasive early detection of DN.

**Supplementary Information:**

The online version contains supplementary material available at 10.1186/s13098-022-00864-3.

## Introduction

Diabetic nephropathy (DN) is the most common microvascular complications (MC) of diabetic patients, which is the major cause of end-stage renal disease (ESRD) [1]. Although comprehensive interventions, including lifestyle changes and drug therapy, can significantly prevent or delay the onset of microvascular complications, these treatments must be conducted as early as possible. However, many diabetic patients with early microvascular damage do not have any symptoms for a long time. Thus, annual screening of microvascular complications is recommended by clinical practice guidelines. Unfortunately, some screening tests need specific conditions, such as mydriatic tests and 24-h urine microalbumin collection, which further compromises patient compliance.

Microangiopathy is the basis of DN, which is associated with low-grade inflammation, epigenetic mechanisms, and the activation of the renin–angiotensin system [2, 3]. The violent fluctuations in blood glucose level were harmful to vascular endothelial function that caused vasodilatation decline and hypoperfusion [4]. In addition, progressive rarefaction of the microvascular network was found in diabetic patients [5]. Sublingual microcirculation was found to be capable of reflecting the situation of visceral microcirculation [6, 7] and has been widely used in the monitoring of microcirculation in critically ill patients to guide treatment [8–10]. Several studies have found that the density of sublingual vasculature changes in diabetes patients [11], and nail capillary alterations in diabetes patients are correlated with retinopathy [12]. However, it is unknown whether sublingual microcirculation can be used as an early screening test for the evaluation of DN in diabetic patients.

In the present study, we investigated the sublingual microcirculation through multi-position peripheral vessel side-stream dark-field (SDF) microcirculation microscopy in healthy subjects and in diabetic patients with and without microvascular complications. We aimed to explore whether sublingual microcirculation can be a window for the assessment of early changes in DN in diabetes.

## Methods

### Study design and participants

This was a cross-sectional observational study performed at Daping Hospital, Army Medical University, Chongqing, China. A total of 100 patients with type 2 diabetes mellitus (T2DM) who were diagnosed based on the WHO diagnostic criteria and 50 healthy subjects were recruited between January 2019 and September 2020. All the participants were aged 18–70 years. Some participants were excluded because of failed imaging capture and analysis, a lack of sufficient laboratory data, or significantly reduced renal function, which may have caused extra bias in the microcirculation evaluation. Finally, 89 T2DM patients and 41 healthy subjects completed the study and were included in the data analysis. This study was conducted according to the principles of the Declaration of Helsinki. All the participants signed informed consent forms before the initiation of any trial-related activities, and the protocol of the study was approved by the ethics committee at Daping Hospital. This study was registered on *ClinicalTrials.gov* (NCT04403945).

Enrollment criteria are patients aged 18–70 years old with T2DM who have been screened for DN, diabetic retinopathy (DR) and diabetic peripheral neuropathy (DPN). Clinical diagnosis of early DN: collected 24-h urine and urine microalbumin creatinine ratio (urinary albumin excretion, UACR) ≥ 30 mg/g without decline in renal function. Fever, refractory hypertension, severe exercise, congestive heart failure, urinary tract infection and other influencing factors must be excluded [13]. Fluorescein fundus angiography was used, and at least two professional ophthalmologists read the film to diagnose DR; the diagnostic criteria met the international standards unified by the American Academy of Ophthalmology in 2002 Criteria for the severity of clinical diabetic retinopathy. Diagnostic criteria of DPN: Abnormal temperature sensation, vibration sensation or abnormal pressure sensation detected by vibration perception testing, 10 g (Semmes–Weinstein) monofilament examination. Nerve conduction velocity reduction confirmed by neuroelectrophysiology and neuropathy caused by other reasons were excluded [14]. The exclusion criteria included the following: (1) T2DM with acute diabetic complications, such as ketoacidosis; (2) type 1 diabetes; (3) alcohol abuse, mental illness, and psychoactive substance abuse; and (4) oral diseases influencing sublingual microcirculation measurement, including periodontal diseases, oral cancers, mouth ulcers, etc.; (5) pregnant or lactating; (6) unwillingness to provide informed consent. Healthy volunteers were screened and recruited for the study by the investigators, who carefully evaluated their medical history, clinical status, and laboratory tests.

### Sublingual microcirculation acquisition and preliminary image data analysis

All participants were asked to sat in the examining room at a temperature of approximately 24 °C and were informed not to consume caffeine-containing substances or any drugs affecting vascular perfusion 2 h prior to the assessment. A multi-position peripheral vessel side-stream dark-field (SDF) hand-held imaging device equipped with a Chinese-Advanced Vessel Analysis-Contrast (C-AVA-C) system (LiHua Xinghuai, XuZhou LiHua Electronic Technology Development Co., Limited, China) was applied to acquire and analyze sublingual microcirculation images. The device is equipped with a hand-held probe fitted with a 5× objective lens system to collect video. The probe is covered by an aseptic transparent lens cover that is disposable. Light from the probe is emitted by a group of centrally placed light-emitting diodes (LEDs). The LED emits stroboscopic green light with a wavelength of 530 nm, which can pass through leukocytes and be absorbed by hemoglobin ignorant of its oxygenation state. In the transmitted image, erythrocytes are darker, while leukocytes seem to be brighter than the illuminated background tissue. As a result, even if the wall ofthe microvessel is not visible, vessel flow can be seen clearly. Representative images in each group are shown in Additional file 1: Fig. S1. To avoid inter-observer bias, the acquisition of images of subjects was conducted by a fixed clinical researcher who was trained to recognize and avoid stress and motion artifacts. Before analysis and processing of the image data, the analyzer was blinded to the participants’ information and disease groups. After gently wiping off oral secretions with cotton swabs, the researcher placed the probe on both sides of the glossodesmus and adjusted the position and focal length of the probe to avoid the effect of pressure on microcirculation to obtain a clear and stable image.

The CVA-C system captured sublingual microcirculation dynamic video and stabilized it to facilitate the observation of vascular velocity; at the same time, it semi-automatically identified the vascular trajectory and generated a report, which included the indicators mentioned in the consensus assessment of microcirculation [9, 15]. In this study, 2–3 video images of at least 20 s each were collected on both sides of the frenulum (if the image quality was not up to the requirements, at least 3 images were retained). We separated the vessels according to their size, with small vessels defined as < 20 µm in diameter. Obstruction of blood flow in larger venules can be used to determine image quality, as it is agreed that the slow flow in these large vessels is due to excessive pressure. After slightly withdrawing the probe and reducing the pressure, the recovery of blood flow was observed. According to the consensus assessment of microcirculation, the occurrence of artifacts was one of the main factors affecting parameter analysis, and we tried to reduce artifacts by observing the blood flow of larger microvessels [15]. Analysis was limited to microcirculation with a diameter of less than 20 µm. We calculated scores concerning microcirculation parameters, including total vessel density (TVD, mm/mm^2^), perfused vessel density (PVD, mm/mm^2^) and proportion of perfused vessels (PPV, %), which were described in detail about the measurement method, significance, and advantages of these indexes in the consensus on the assessment of sublingual microcirculation [9]. Among these parameters, as determinants of capillary distance (diffusive capacity) and red blood cell velocity (convective capacity), PVD is regarded as the gold standard in preclinical research.

### Biochemical and anthropometric measurements

Fasting plasma glucose (FPG), HbA1c, total cholesterol (TC), triglycerides (TGs), high-density lipid-cholesterol (HDL-c), low-density lipid-cholesterol (LDL-c), alanine aminotransferase(ALT), aspartate aminotransferase(AST), blood urea nitrogen(BUN), and estimated glomerular filtration rate(eGFR) were measured after overnight fasting for 8 h. Anthropometric measurements, including blood pressure, waist circumference, height, and body weight, were performed by trained assessors. Office blood pressure measurements [16] were performed on both arms in the sitting position after 10 min of rest. The subject was seated comfortably with the back supported and the upper arm bare without constrictive clothing. Cuffs of the appropriate size were placed around the subject’s arm. The mercury column was slowly deflated at 2 to 3 mm/s, and the first and last audible Korotkoff sounds were taken as systolic and diastolic blood pressure, respectively. Three readings were taken at intervals of 2 min, and the average value was used as the participant’s blood pressure. BMI was defined as body weight (kg) divided by the square of body height (meter).

### Screening and clinical diagnosis of diabetes microvascular complications

All the diabetes patients were screened for microvascular complications under the instruction of guidelines [17], and all the patients had undergone ophthalmological consultation and fundus examination. Participants were given both verbal and written instructions on how to perform the 24-h urinary collection. Each participant was provided with a urine collection bottle (50 ml) filled with 5 ml formaldehyde solution, a polypropylene graduated cylinder with a capacity of 1000 ml, a plastic cask (4000 ml), and a black plastic bag for carrying the bottle. On the first morning of the urine collections, the participants were instructed to discard the first specimen and then to collect all specimens for up to 24 h, up to and including the first specimen the following day. Urine volume was recorded by the assessors.

### Statistical analyses

The results are presented as the mean ± standard deviation, median with interquartile range, or n (%). The Kolmogorov–Smirnov test or the Shapiro–Wilk test was used to determine whether each variable had a normal distribution. The baseline characteristics of patients were compared between groups using the chi-squared test for categorical variables, one-way analysis of variance (ANOVA) with Games–Howell multiple comparison post hoc test for continuous variables with normal distribution, and the K-sample Kruskal–Wallis one-way ANOVA nonparametric test for data with non-normal distribution. Spearman’s non-parametric correlation analysis was performed to assess the relationship between microcirculatory parameters, urinary microalbumin creatinine ratio, fasting blood glucose, and HbA1c. Sensitivity and 1-specificity for different cutoffs of the TVD and PVD were plotted in receiver operating characteristic (ROC) curves. The optimal cutoff point was assessed using Youden’s J statistic. Numerical statistical analyses were conducted using SPSS software, version 23.0 (SPSS, Inc.) and OriginPro software, version OriginPro 2021b (OriginLab Co.), and a two-sided *P* < 0.05 was considered statistically significant.

## Results

### Baseline characteristics of the participants

**Fig. 1:**
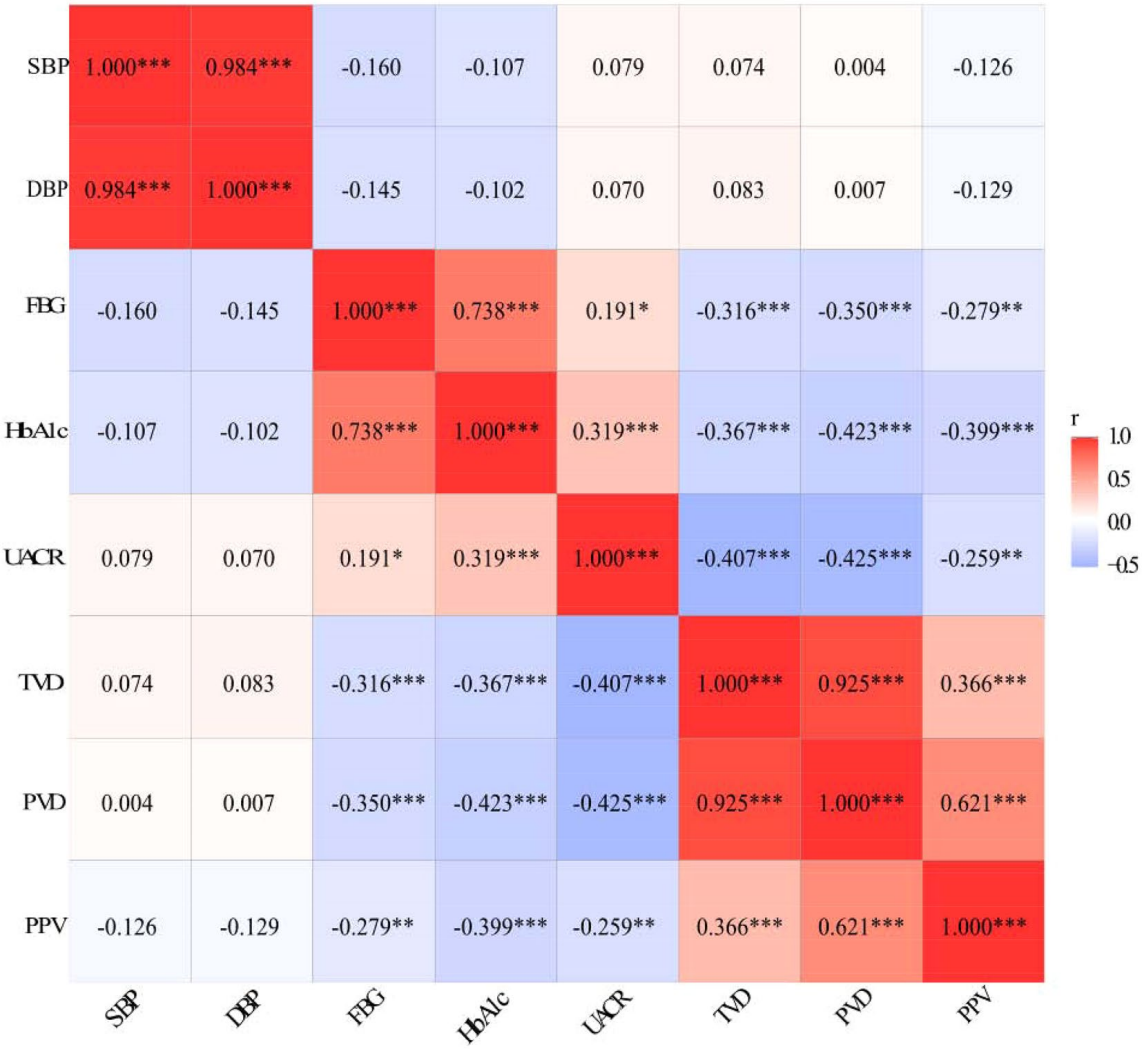
Correlation of microcirculatory parameters with metabolic indexes and UACR. SBP: systolic blood pressure; DBP: diastolic blood pressure; FBG: fasting blood glucose; UACR: urinary albumin creatinine ratio. Spearman correlation analysis was used. Significant values are shown. Orange and blue colors represent significant positive correlations and negative correlations, respectively. Darker color represents stronger correlations

The baseline characteristics of the participants are summarized in Table 1. Of the 89 diabetes patients, 44 were screened positive for MC [38 (86.36%) with DN, 33 (75%) with neuropathy, and 21 (47.73%) with retinopathy]. No significant differences in age, sex, BMI, SBP, DBP, AST, ALT, TC, TG, BUN or LDL-c were observed among the three groups. Diabetic patients with microvascular complications had a longer disease duration, higher FPG, larger HbA1c, and increased UACR. We calculated three widely used microcirculatory parameters, TVD, PVD and PPV, which showed high consistency with blood glucose levels (FPG: TVD, r =  − 0. 316, *P* < 0.001; PVD, r =  − 0. 350, *P* < 0.001; PPV, r =  − 0. 279, *P* = 0.001; HbA1c: TVD, r =  − 0. 367, *P* < 0.001; PVD, r =  − 0. 423, *P* < 0.001; PPV, r =  − 0.399, *P* < 0.001) (Fig. 1). This suggests that hyperglycemia dominantly jeopardizes the sublingual microcirculation in diabetes patients.

**Table 1:** Baseline characteristics of the participants in each group

	Control (n = 41)	DM (n = 45)	DM_MC (n = 44)
Age, year	54.88 ± 9.09	55.47 ± 6.22	55.25 ± 8.28
Male sex, no. (%)	22 (53.66)	29 (64.44)	27 (61.36)
Duration of DM, year	Na	5.76 ± 4.36	11.45 ± 5.55***
Body-mass index, kg/m^2^	24.64 ± 2.33	24.05 ± 2.14	24.25 ± 3.76
Systolic blood pressure, mmHg	123 ± 10	121 ± 10	122 ± 7
Diastolic blood pressure, mmHg	75 ± 10	74 ± 10	74 ± 7
Fasting blood glucose, mmol/L	4.82 ± 0.49	8.88 ± 3.38***	9.63 ± 4.07***
HbA1c, % (mmol/mol)	5.46 ± 0.52 (37)	10.21 ± 2.81 (88)***	10.48 ± 2.12 (91)***
AST, u/L	26.32 ± 11.86	21.76 ± 8.54	21.85 ± 12.93
ALT, u/L	22.33 ± 14.49	23.84 ± 10.77	23.12 ± 18.94
TC, mmol/L	4.25 ± 0.67	3.84 ± 1.57	3.61 ± 1.63
TG, mmol/L	1.41 ± 0.44	1.94 ± 1.40	1.68 ± 1.07
HDL-c, mmol/L	1.30 ± 0.27	1.07 ± 0.23***	1.01 ± 0.24***
LDL-c, mmol/L	2.89 ± 0.79	2.93 ± 0.67	2.79 ± 0.72
UACR, mg/g.Cr	13.09 [8.87, 13.73]	8.36 [5.93, 15.42]	185.10 [45.95, 558.1]***^###^
Crea, umol/L	53.38 ± 9.41	57.72 ± 12.74	61.33 ± 14.74*
BUN, mmol/L	5.69 ± 1.66	5.78 ± 1.27	6.13 ± 1.96

Data are mean ± SD, median with inter-quartile range, or n (%). The body mass index is the weight in kilograms divided by the square of the height in meters

*SD* standard deviation, *HbA1c* glycated hemoglobin, *TC* total cholesterol, *TG* triglycerides, *HDL-c* high density lipid-cholesterol, *LDL-c* low density lipid-cholesterol, *MC* microvascular complications, *ALT* alanine aminotransferase, *AST* aspartate aminotransferase, *BUN* blood urea nitrogen, *eGFR* estimated glomerular filtration rate

*P < 0.05, ***P < 0.001 vs. control group; ^###^*P* < 0.001 vs. DM group

### Dysfunction of sublingual microcirculation in diabetes patients with microvascular complications

Representative images of sublingual microcirculation from each group are shown in Additional file 1: Fig. S1. In the control group, there was no single red blood cell stasis, the boundary was smooth, and the microcirculatory network was rich and uniform. In contrast, the sublingual blood vessel density was decreased in diabetes patients, and siltation of erythrocytes was seen in the microcirculation. The boundary of the vessels was interrupted, and single red blood cell imaging could be seen. Importantly, the vascular density was significantly reduced in diabetes patients with microvascular complications, accompanied by the more obvious detour and stiffness of blood vessels and the proportion of vascular stasis.

To make a quantitative comparison of the above differences, we further calculated the sublingual microcirculatory parameters from each group. Diabetic patients presented insufficient microcirculation perfusion compared with healthy controls (Table 2), and reductions in TVD (9.67 ± 1.94 vs. 10.89 ± 2.16 mm/mm^2^, *P* < 0.05), PVD (8.64 ± 2.46 vs. 10.30 ± 2.27 mm/mm^2^, *P* < 0.001), and PPV (0.92 [0.84, 0.98] vs. 1.00 [0.92, 1.00]%, *P* < 0.001) were observed. In further research, it was found that compared with patients without MC, diabetic patients with MC presented with more severe reductions in TVD (7.07 ± 1.64 vs. 9.67 ± 1.94 mm/mm^2^, *P* < 0.001) and PVD (5.88 ± 1.82 vs. 8.64 ± 2.46 mm/mm^2^, *P* < 0.001). As expected, diabetes patients with microvascular complications had dramatically decreased microcirculatory parameters compared with the control group (*P* < 0.001).

**Table 2:** Comparison of sublingual microcirculatory parameters in each group

	Control (n = 41)	DM (n = 45)	DM_MC (n = 44)
TVD, mm/mm^2^	10.89 ± 2.16	9.67 ± 1.94*	7.07 ± 1.64***^###^
PVD, mm/mm^2^	10.30 ± 2.27	8.64 ± 2.46***	5.88 ± 1.82***^###^
PPV, %	1.00 [0.92, 1.00]	0.92 [0.84, 0.98]***	0.87 [0.76, 0.95]***

*TVD* total vascular density, *PVD* perfused vessel density, *PPV* proportion of perfused vessel, *MC* microvascular complications

*P < 0.05, ***P < 0.001vs. control group; ###*P* < 0.001 vs. DM group

### Early screening of DN using sublingual microcirculation

Since the most common MC in this study was DN (86.36% of the diabetic patients), we next tried to explore the feasibility of using sublingual microcirculation for early DN detection. The UACR was found to be closely correlated with the sublingual microcirculation; the more severe the damage to the sublingual microcirculation was, the higher the UACR level was in these patients (TVD, r =  − 0.407, *P* < 0.001; PVD, r =  − 0.425, *P* < 0.001; PPV, r =  − 0.259, *P* = 0.002) (Fig. 1). Then, we calculated the receiver operating characteristic (ROC) curve and the area under the curve (AUC) of TVD and PVD for the early screening of DN. ROC curve analysis showed that both the TVD (AUC = 0.890 [0.836 0.944], *P* < 0.001) and PVD (AUC = 0.883 [0.826, 0.940], *P* < 0.001) could be valuable indicators for the screening of DN. Using the Youden index (YI), we calculated that the best tangent point of TVD was 9.08 mm/mm^2^ (sensitivity: 90.9%, specificity: 70.9%) and that for PVD was 7.54 mm/mm^2^ (sensitivity: 81.8%, specificity: 80.2%). In an attempt to improve the AUC curve of the above microcirculatory parameters, we tried to use binary logistic regression analysis to obtain a combined predictor. The regression equation was:

$$logit\left( {group} \right) = 7.652-0.803 \times TVD - 0.196 \times PVD$$

We removed the constant item, and the combined predictor index (CPI) was:

$${\text{index}} = TVD + {\raise0.7ex\hbox{${0.196 \times PVD}$} \!\mathord{\left/ {\vphantom {{0.196 \times PVD} {0.803}}}\right.\kern-\nulldelimiterspace} \!\lower0.7ex\hbox{${0.803}$}}$$

**Fig. 2:**
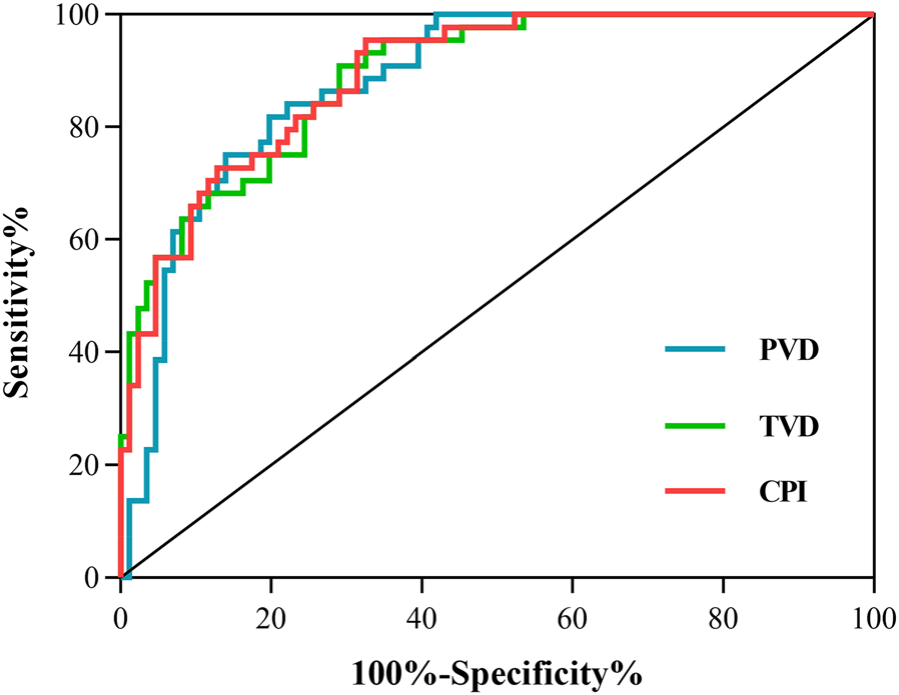
ROC curve of TVD, PVD and CPI in diagnosing DN

Using the CPI, we found that both the AUC (0.892, [0.838 0.945], *P* < 0.001) and cutoff point of 11.30 mm/mm^2^ showed great improvement (sensitivity: 95.5%, specificity: 67.4%). The results are illustrated in Fig. 2 and Additional file 1: Table S1. To further verify their efficiency in screening early DN, we recruited another 39 hospitalized diabetic patients and performed sublingual microcirculation monitoring. We found strong agreement using Cohen’s kappa statistic (TVD, κ = 0.647, 95% CI 0.418–0.876, *P* < 0.001; PVD, κ = 0.896, 95% CI 0.755–1.037, *P* < 0.001; CPI, κ = 0.651, 95% CI 0.431–0.871, *P* < 0.001).

## Discussion

In this study, we established method for the early detection of DN in diabetic patients by monitoring the sublingual microcirculation. We found that sublingual microcirculation status was closely related to blood glucose, which was significantly damaged in diabetes patients compared with healthy participants. We also showed that microcirculatory parameters, TVD and PVD, were remarkably decreased in diabetes patients with MC. Both TVD and PVD were highly associated with UACR levels, which showed great sensitivity in screening DN. To further optimize the analysis indicators, we obtained a CPI to better perform the early assessment of diabetic microvascular disease. These results were further validated in diabetic patients with definite nephropathy.

Diabetic microangiopathy is characterized by vascular endothelial dysfunction in the early stage (1). Microcirculation monitoring has been widely studied in severe infections, including sepsis, dengue, and influenza [18–20]. It has been shown to be effective in predicting disease outcomes and helping therapeutic adjustment. Microcirculation changes in the sublingual tissue of patients with sepsis were strongly correlated with the severity of the disease [21]. A continuous hyperglycemic environment could lead to endothelial dysfunction, reduce blood flow and cause atrophy of the microvascular network, thereby aggravating tissue hypoxia and leading to diabetic microvascular complications [22]. Because of the various demands of oxygen consumption of the organs, microcirculatory lesions may also present differently. It has been reported that different tissues (retina, ear lobe, and cremaster muscle) exhibit similar microcirculatory lesions in diabetic mice [23]. In addition, capillary recruitment of skeletal muscle is impaired in diabetic patients with MC [24]. In our study, we provided in vivo evidence that the status of the sublingual microcirculation was closely related to hyperglycemia in diabetic patients.

Diabetic patients had decreased glycocalyx density compared with healthy controls [25]. We found that sublingual blood vessel density was decreased in diabetes patients, and microcirculatory parameters, including TVD and PVD, were significantly decreased. In contrast to our findings, Wadowski et al. showed that the perfused and total capillary density showed no difference between diabetes patients and healthy controls. The reasons for these discrepancies may be as follows: (1) the relatively small sample size and mixed types of diabetes patients in Wadowski’s study; (2) diabetes patients had higher blood glucose and HbA1c levels in our study, which might present earlier damage to the microcirculation; and (3) different analysis methods and manual measurement of the microvessels. Furthermore, we found that the microcirculatory parameters were closely correlated with the marker of renal dysfunction, UACR, which is an indicator of the early stage of DN. Although the annual measurement of spot UACR was recommended, the existing individual variability made it necessary to complete at least three sample collections within 6 months or to perform the 24-h urinary collection [26]. Thus, many diabetes patients who develop early nephropathy might be lost to follow-up or unable to finish the examination. In recent years, miRNAs have been reported to have substantial potential as biomarkers for vascular complications of T2DM [27]. However, there is no clear evidence regarding which kind of miRNA is best for early warning of complications, and there is a lack of a standardized method to verify the results. In this study, we found that the microcirculatory parameters TVD and PVD could be valuable indicators for the screening of DN using the noninvasive measurement of sublingual microcirculation. We further validated its diagnostic value in 39 hospitalized diabetic patients with DN, which showed great consistency.

Annual screening of diabetic retinopathy and neuropathy was more difficult to achieve, since most of the patients would not represent typical signs and symptoms at an early stage. There are several methods like color fundus photos, fluorescein angiography and OCT angiography can be used for screening diabetic microangiopathy. However, these methods always need mydriatic or invasive procedures, and patients needed to visit the eye clinic to undergo special examinations, which will compromise patient compliance and delay the diagnosis of diabetic retinopathy [28]. Vibration perception testing using a 128 Hz tuning fork together with a 10 g (Semmes–Weinstein) monofilament was used for the screening of neuropathy, while it can only detect neuropathy at a late, irreversible stage [29]. In our work, using SDF-derived sublingual microcirculation monitoring, we successfully detected an early reduction in microcirculation in diabetes patients compared with healthy controls. Therefore, this method might also have the potential for early and noninvasive screening diabetic retinopathy and neuropathy, which need to be further tested in future studies.

There are certain limitations in this study. This is an observational study, and further prospective and diagnostic accuracy studies with larger sample sizes need to be performed. Evaluation of vascular perfusion still depends on artificial recognition, which might cause bias between operators, and computation-based automated analysis should be developed.

Overall, we established a method of using sublingual microcirculation to evaluate diabetes microangiopathy. This method showed good sensitivity in diagnosing DN. Through future verification in a larger population sample, this method is expected to become a noninvasive screening method for early DN.

## Supplementary Information

**Additional file1: Figure S1.** Representative SDF images of microcirculation density and perfusion in the three groups. **Table S1.** Diagnostic value of microcirculatory parameters in diabetic patients.

## Data Availability

The data used to support the findings of this study are available from the corresponding author on reasonable request.

## Abbreviations

T2DM: Type 2 diabetes mellitus
HbA1c: Glycated hemoglobin
DR: Diabetic retinopathy
DN: Diabetic nephropathy
DPN: Diabetic peripheral neuropathy
MACEs: Major adverse cardiovascular events
TVD: Total vascular density
PVD: Perfused vascular density
PPV: Proportion of perfused vessels
UACR: Urinary albumin creatinine ratio
CPI: Combined predictor index
