# Supplementary material for: Assessment of sublingual microcirculation for the screening of diabetic nephropathy
Source: Diabetol Metab Syndr. 2022 Jul 6;14:90. doi: 10.1186/s13098-022-00864-3 (PMC9258215; doi:10.1186/s13098-022-00864-3)
Supplement: Supplementary file 1 — Additional file1: Figure S1. Representative SDF images of microcirculation density and perfusion in the three groups. Table S1. Diagnostic value of microcirculatory parameters in diabetic patients. [file 13098_2022_864_MOESM1_ESM.docx]

**Additional MATERIAL**

**Assessment of sublingual microcirculation for the screening of diabetic nephropathy**

**This file includes:**

Figure S1. Representative SDF images of microcirculation density and perfusion in the three groups.

Table S1. Diagnostic value of microcirculatory parameters in diabetic patients.


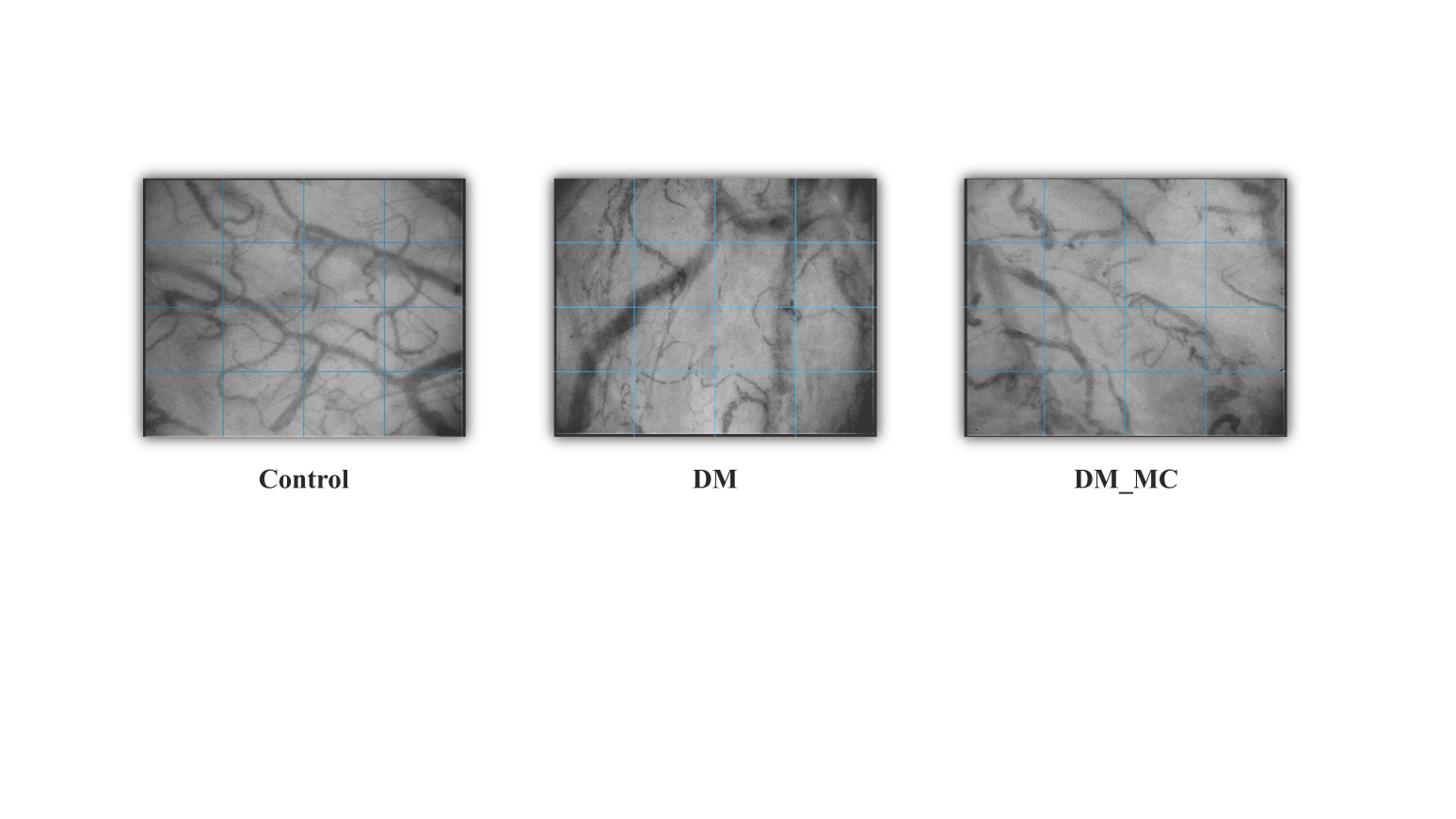


**Figure S1. Representative SDF images of microcirculation density and perfusion in the three groups.** In the control group(a), because of the fast flow rate, no single red blood cell could be seen, with a smooth boundary. In the diabetes group(b),wefound that the blood vessel density was decreased, most of the red blood cell stasis existed in the microvessels, the boundary was intermittent, and a single red blood cell imaging could be seen. Furthermore, for diabetic patients with MC(c), the sublingual vascular density was further decreased, tortuosity and rigidity were more obvious, and the proportion of stagnant blood vessels increased.

|  | Optimal threshold | AUC | *P* value | Sensitivity | Specificity | YI |
| --- | --- | --- | --- | --- | --- | --- |
| TVD — mm/mm^2^ | 9.07615 | 0.890 | <0.001 | 0.909 | 0.709 | 0.618 |
| PVD — mm/mm^2^ | 7.53665 | 0.883 | <0.001 | 0.818 | 0.802 | 0.620 |
| CPI — mm/mm^2^ | 11.2997 | 0.892 | <0.001 | 0.955 | 0.674 | 0.629 |

**Table S1. Diagnostic value of microcirculatory parameters in diabetic patients.** AUC: area under the ROC curve; YI: Youden index.
